# Diagnoses of Multiple Sclerosis and Related Disorders and Disease‐Modifying Therapies: A Comparison of the Danish Multiple Sclerosis Registry With Other Danish Health Registries

**DOI:** 10.1002/brb3.71235

**Published:** 2026-02-10

**Authors:** Hanna Joensen, Elisabeth Framke, Luigi Pontieri, Melinda Magyari

**Affiliations:** ^1^ The Danish Multiple Sclerosis Registry, Department of Neurology Copenhagen University Hospital ‐ Rigshospitalet Glostrup Denmark; ^2^ Danish Multiple Sclerosis Center, Department of Neurology Copenhagen University Hospital ‐ Rigshospitalet Glostrup Denmark; ^3^ Department of Clinical Medicine University of Copenhagen Copenhagen Denmark

**Keywords:** diagnoses, disease‐modifying treatment, multiple sclerosis, nationwide, registries

## Abstract

**Objectives:**

Comparison of recorded diagnoses of multiple sclerosis (MS) and related disorders and disease‐modifying therapies (DMTs) in The Danish Multiple Sclerosis Registry (DMSR) with other nationwide health registries. The aim of the study is to describe and compare information on diagnoses of MS and related disorders and treatments with DMTs available in three national registries, highlighting the key differences relevant to MS research and providing insight for researchers for their choice of data source(s) suitable for their study.

**Materials and Methods:**

DMSR is a disease registry encompassing information on persons with MS and related disorders. The Danish National Patient Registry (DNPR) is a registry of activities at Danish hospitals. The Danish National Hospital Medication Registry (DNHMR) contains information on in‐hospital prescription medications. The population comprised all persons in DMSR in 2023 who were alive or born after and residing in Denmark on January 1, 1995 (N = 26,474). For this population, we identified DNPR contacts with diagnoses of MS or related disorders and initiated DMTs in DNHMR. Diagnostic and demographic characteristics were reported as recorded in DMSR for the total population, and the subset included in DMSR but not in DNPR. Characteristics of the part of the population identified in DNPR were reported as recorded in DNPR. We calculated the proportions of DMT treatments in DMSR identified in DNHMR.

**Results:**

Of the 26,474 persons, 23,857 (90.1%) were recorded with a diagnosis of MS or a related disorder in DNPR. Most (86.6%) of the 2617 persons not identified in DNPR were diagnosed before 1995. The proportion of persons with MS recorded without specification of the disease phenotype was 23.5% in the DMSR and 76.2% in the DNPR. After 2005, only 1.8% of persons with MS were recorded with an unspecified phenotype in DMSR. Of a total of 18,168 initiated DMT treatments in DMSR, 7230 (39.8%) were identified in DNHMR, with proportions ranging from 0.0% (mitoxantrone) to 88.5% (ocrelizumab).

**Conclusions:**

DMSR is suitable for disease‐specific research addressing treatment efficacy, disease development, and long‐term outcomes. In contrast, DNPR is well suited for broad epidemiological studies involving various health conditions and hospital utilization. The DNHMR will, when matured, be useful for studies involving medical treatment and comedication. However, as each registry has limitations, for most studies the best approach will be combining registries, depending on the research question.

## Graphical abstract

This study aimed to describe and compare information on diagnoses of MS and related disorders as well as treatments with disease‐modifying therapies (DMTs) available in three national registries, highlighting the key differences relevant to MS research. The Danish Multiple Sclerosis Registry (DMSR) is a disease registry encompassing a wide range of information on persons with MS and related disorders established in 1956. 26,474 people with MS or related disorders, 24,552 people with MS, 76.5% with specified phenotype, 18,168 treatments involving 11,602 people with MS, The Danish National Patient Registry (DNPR) is a registry of activities at Danish hospitals established in 1977. 87.7% of the people with MS or related disorders in DMSR were identified with the same primary diagnosis, 92.4% of the people with MS in DMSR were identified with an MS diagnosis, and 23.8% with a specified phenotype. The Danish National Hospital Medication Registry (DNHMR) contains information on in‐hospital prescription medications established in 2018. 7230 treatments involving 5944 persons (39.8%) were identified compared to DMSR.

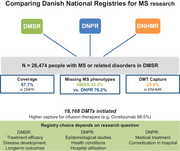

## 1 Introduction

Denmark's nationwide extensive health registries have supported a long tradition of epidemiological research (Laugesen et al. 2021, Schmidt et al. 2019). When planning a study on a certain disease, such as multiple sclerosis (MS), researchers should select the most suitable data source for answering the scientific question. The Danish National Patient Registry (DNPR) (Schmidt et al. 2015), the Danish National Hospital Medication Registry (DNHMR) (The Danish Health Data Authority n.d.), and the Danish Multiple Sclerosis Registry (DMSR) (Magyari et al. 2021, Koch‐Henriksen 1999) are data sources that could be used individually or in combination for such purpose.

When comparing DNPR, DNHMR, and DMSR, it becomes obvious that each registry offers different types of information on the individual patient. While DNPR contains data on all hospital contacts, including the purpose of the contact (coded using the International Classification of Diseases 10th and 8th revisions [ICD‐10; ICD‐8]), specific medical treatments, surgical procedures, and examinations (Schmidt et al. 2015), the DNHMR contains only data on prescriptions of medications dispensed at hospitals (The Danish Health Data Authority n.d.). DMSR provides a broader range of longitudinal clinical and demographic information about persons with MS from clinical onset and throughout the disease development (Magyari et al. 2021). All contacts at all MS clinics in Denmark are recorded, along with expanded disability status scale (EDSS) (Kurtzke 2015) scores during the clinical visit, results from magnetic resonance imaging (MRI) scans, relapses, disease‐modifying treatment (DMT) history, and adverse events associated with DMTs.

This study aimed to describe and compare information on diagnoses of MS and related disorders and treatments with DMTs available in these three national registries, highlighting the key differences relevant to MS research.

## 2 Materials and Methods

### 2.1 Data Sources

#### 2.1.1 The Danish Multiple Sclerosis Registry

DMSR is a nationwide population‐based disease registry encompassing comprehensive demographic and clinical information on persons with MS and related disorders.

The registry was founded in 1956 following a nationwide prevalence survey of MS in Denmark in 1949 and a continuous registration of incident MS cases since 1948. Since then, DMSR has been updated and monitored by neurologists based on clinical records on all persons diagnosed with MS and related disorders in Denmark with the purpose of collecting valid data suitable for research. Until 2015, discharge letters or copies of clinical records were sent to the DMSR, and the diagnosis was validated by a few dedicated neurologists. In 2015, this procedure for data entering into the registry was replaced by an online data collection platform (Magyari et al. 2021) shared with the Clinical Quality Database, The Danish Multiple Sclerosis Treatment Register (DMSTR) (Magyari et al. 2016, Koch‐Henriksen et al. 2015), established in 2007 by the Danish Health Authorities to monitor the quality of MS treatment in Denmark through a number of quality indicators.

DMTs for the treatment of relapsing remitting multiple sclerosis (RRMS) became available in Denmark in 1996. There are 13 MS clinics in Denmark operated by public hospitals, which are the only ones allowed to diagnose MS, and to prescribe and dispense DMTs. Neurologists at the MS clinics are entering data into the online platform constituting the DMSR. Since 1996, it has been mandatory to monitor all persons treated with DMTs at least once or twice a year, depending on the DMT, and to report data on DMT use, and MS disease activity. Treating neurologists from all MS clinics in Denmark are joined in a network, the Danish Multiple Sclerosis Group (DMSG), meeting twice a year to discuss and establish uniform guidelines for MS care. From this follows a virtually complete and regular registration of clinical disease activity. Quality control and assurance measures are taken on a regular basis, securing complete, consistent, and valid data.

#### 2.1.2 The Danish National Patient Registry

DNPR, established in 1977, is a nationwide register of activities at Danish hospitals. DNPR encompasses administrative and clinical data on persons discharged from Danish non‐psychiatric hospitals since 1977, and on psychiatric inpatients, emergency department, and outpatient contacts since 1995. Information in DNPR includes diagnoses, certain medical treatments, surgical procedures, and examinations (Schmidt et al. 2015, The Danish Health Data Authority n.d.).

#### 2.1.3 The Danish National Hospital Medication Register

DNHMR (The Danish Health Data Authority n.d.) is a newly established registry. The reporting of data to the registry started in 2018, and the registry is not yet complete. DNHMR contains selected information on certain in‐hospital prescription medications in public hospitals, coded using the Anatomical Therapeutic Chemical Classification (ATC) nomenclature, prescription date, administration date, and diagnoses associated with the medication (i.e., indications). Recorded data are retrieved from the electronic medicine module of the hospital's region. The purpose of DNHMR is drug safety surveillance, drug utilization, research, and clinical quality assurance.

### 2.2 Study Population

We included all persons registered in DMSR at the data extraction date, November 8, 2023. By linking this population to the Danish Civil Registration System (CRS) (Pedersen 2011), we excluded persons not residing in Denmark and persons who emigrated or died before January 1, 1995. The study population consisted of 26,474 persons with MS or related disorders who were alive or born after and residing in Denmark on January 1, 1995.

### 2.3 Diagnoses

In DMSR, diagnoses are recorded as MS, CIS (clinically isolated syndrome), RIS (radiologically isolated syndrome), NMOSD (neuromyelitis optica spectrum disease), and MOGAD (myelin oligodendrocyte glycoprotein antibody‐associated disease). Persons diagnosed with MS are further classified by phenotype at the date of diagnosis (i.e., relapsing remitting MS), primary progressive MS, secondary progressive MS, or unspecified MS. For those transitioning from relapsing remitting to secondary progressive MS, the date of transition is also recorded.

For the study population of 26,474 persons with MS or related disorders, we identified all contacts in DNPR from 1995 until December 31, 2023, with a primary diagnosis (main reason for the hospital contact) or secondary diagnosis (supplement to primary diagnosis) of MS or related disorder (CIS, NMOSD, MOGAD, or RIS) based on ICD‐10 codes (G35 including subcodes and Z033C (observation for MS), G360, G369, G378, and G379). The diagnostic criteria, the changes in diagnostic criteria, and the time point of change in diagnostic criteria during the study period are affecting DMSR and DNPR identically.

### 2.4 Disease‐Modifying Therapies

In DMSR, treatments with DMTs are documented by both brand and generic name along with the start and stop dates of the treatments. Pulse treatments are further recorded with a course number and start and stop dates for each course. The reason for discontinuing a DMT is recorded, as well as reasons for not receiving DMT. For DMTs with multiple administration modes, they are recorded by type and change in administration mode.

For the study population of 26,474 persons with MS or related disorders, we identified DMTs prescribed for MS in DNHMR using the ATC codes shown in Table 1, with a prescription date from January 1, 2018, to November 8, 2023, while excluding treatments recorded in DNHMR that were associated with a diagnosis other than MS.

**TABLE 1:** ATC codes included to identify DMTs in DNHMR.

DMT	ATC code
Alemtuzumab	L04AA34
Azathioprin	L04AX01
Cladribine	L04AA40
Dimethyl fumarate	L04AX07
Diroximel fumarate	L04AX09
Fingolimod	L04AA27
Glatiramer acetate	L03AX13
Interferon beta‐1a	L03AB07
Interferon beta‐1b	L03AB08
Methotrexate	L04AX03
Mitoxantrone	L01DB07
Mycophenolatmofetil	L04AA06
Natalizumab	L04AA23
Ocrelizumab	L04AA36
Ofatumumab (Arzerra)	L01FA02
Ofatumumab (Kesimpta)	L04AA52
Ozanimod	L04AA38
Peginterferon beta‐1a	L03AB13
Rituximab	L01FA01
Siponimod	L04AA42
Teriflunomide	L04AA31

Abbreviations: ATC: Anatomical Therapeutic Chemical Classification; DMT: Disease‐modifying treatment, including off‐label treatments used for MS and related disorders; DNHMR: Danish National Hospital Medication Register.

### 2.5 Statistical Analyses

Categorical variables were reported as frequencies and proportions, and continuous variables were reported as median and interquartile range (IQR).

Demographic and clinical characteristics of the study population were reported in terms of diagnosis, year of diagnosis, and age at diagnosis as recorded in DMSR. These characteristics were summarized for the entire study population (N = 26,474), and the subset recorded in DMSR but not in DNPR (N = 2617). Similarly, characteristics were reported for persons recorded in the DNPR with either a primary or secondary diagnosis (N = 23,857) and for persons with a primary diagnosis only (N = 23,227) as recorded in the DNPR (Table 2).

**TABLE 2:** Comparison of DMSR and DNPR for diagnoses of MS and related disorders.

	DMSR	DMSR only ‐ DMSR diagnosis	DNPR primary or secondary diagnosis	DNPR primary diagnosis
**Persons included; N (%)**	26,474 (100.0)	2617 (9.9)	23,857 (90.1)	23,227 (87.7)
**Diagnosis; N (%)**				
RIS (G369)^a^	96 (0.4)	23 (0.9)	9 (0.0)	<=9 (#)
CIS (G379)	1642 (6.2)	983 (37.6)	387 (1.6)	379 (1.6)
MS (G35)	24,552 (92.7)	1597 (61.0)	23,289 (97.6)	22,676 (97.6)
NMOSD (G360)	112 (0.4)	5 (0.2)	41 (0.2)	<=41 (#)
MOGAD (G378)	72 (0.3)	9 (0.3)	15 (0.1)	15 (0.1)
Observation for MS (Z033C)			116 (0.5)	108 (0.5)
**Year, first diagnosis; N (%)**				
−1994	7861 (29.7)	2,266 (86.6)	NA	NA
1995–2004	5531 (20.9)	141 (5.4)	9503 (39.8)	8853 (37.7)
2005–2014	6667 (25.2)	104(4.0)	6989 (29.3)	6905 (29.7)
2015–2023	6415 (24.2)	106 (4.0)	7365 (30.9)	7569 (32.6)
**Age, diagnosis (years); median (IQR)**				
CIS	37.0 (29.2; 45.3)	35.6 (28.8; 44.1)	41.0 (31.8; 52.0)	40.7 (31.6; 51.1)
MS	39.3 (30.7; 48.3)	39.0 (30.3; 47.5)	43.9 (34.1; 53.8)	43.6 (34.0; 53.3)
NMOSD	48.9 (35.4; 61.2)	43.7 (41.8; 53.1)	50.6 (33.0; 64.3)	# (#;#)
MOGAD	37.7 (30.0; 49.6)	31.6 (27.2; 41.0)	37.5 (24.1; 46.8)	37.5 (24.1; 46.8)
RIS	44.8 (35.8; 55.6)	51.1 (36.1; 59.9)	40.1 (35.2; 53.5)	# (#; #)

Abbreviations: DMSR: The Danish Multiple Sclerosis Registry; DNPR: Danish National Patient Registry; IQR: interquartile range; MS: multiple sclerosis; RIS: radiologically isolated syndrome; CIS: clinically isolated syndrome; NMOSD: neuromyelitis optica spectrum disease; MOGAD: myelin oligodendrocyte glycoprotein antibody‐associated disease.

^a^ G369 includes unspecified demyelinating diseases—therefore not suitable for comparison between registers.

In the DNPR, individuals may be recorded over time with different MS and related disorder diagnoses. Consequently, the date of definite MS diagnosis may carry some uncertainty. For individuals with a combination of MS and related disorders diagnoses, diagnoses were prioritized in the following order: MS as the main priority, followed by CIS, NMOSD, MOGAD, and RIS.

For persons diagnosed with MS in the DMSR, additional characteristics were reported, including year of clinical onset, diagnostic delay, disease duration, age at clinical onset, age at diagnosis, and phenotype at the time of diagnosis. These data were summarized for all persons with MS in DMSR (N = 24,552) and for the subset with MS recorded in DMSR but not in DNPR (N = 1597). For those with MS who were also recorded in DNPR with a primary diagnosis (N = 22,676), the same characteristics were reported as recorded in DNPR. However, certain characteristics could not be assessed using DNPR data and are marked as “NA” (Table 3).

**TABLE 3:** Comparison of DMSR and DNPR for diagnoses of MS.

MS diagnosis in DMSR	DMSR	DMSR only ‐ DMSR diagnosis	DNPR—primary diagnosis
**Persons included; N (%)**	24,552 (100.0)	1597 (6.5)	22,676 (92.4)
**Year, MS onset; N (%)**			
−1994	9220 (37.6)	1477 (92.5)	NA
1995–2004	5333 (21.7)	49 (3.1)	NA
2005–2014	5789 (23.6)	38 (2.4)	NA
2015–2023	4210 (17.2)	33 (2.1)	NA
**Year, MS diagnosis; N (%)**			
−1994	6860 (27.9)	1458 (91.3)	NA
1995–2004	5335 (21.7)	53 (3.3)	8686 (38.3)
2005–2014	6368 (25.9)	43 (2.7)	6752 (29.8)
2015–2023	5989 (24.4)	43 (2.7)	7238 (31.9)
**Diagnostic delay (years); median (IQR)**	2.0 (0.4; 6.0)	2.0 (1.0; 7.0)	NA
**Age, onset (years); median (IQR)**	34.3 (26.8; 43.1)	33.1 (26.2; 41.4)	NA
**Age, diagnosis (years); median (IQR)**	39.3 (30.7; 48.2)	39.0 (30.3; 47.5)	43.6 (34.0; 53.3)
**Disease duration (years); median (IQR)**	20.4 (11.4; 31.0)	39.0 (28.0; 50.0)	NA
**Phenotype, MS diagnosis; N (%)**			
RR (G359A)	15,915 (64.8)	75 (4.7)	3911 (17.3)
PP (G359B)	2481 (10.1)	16 (1.0)	334 (1.5)
SP (G359C)	394 (1.6)	<5 (#)	1152 (5.1)
Unspecified (G35(9))	5762 (23.5)^a^	# (#)	17,279 (76.2)^b^

Abbreviations: DMSR: The Danish Multiple Sclerosis Registry; DNPR: Danish National Patient Registry; IQR: interquartile range; MS: multiple sclerosis; RR: relapsing remitting; PP: primary progressive; SP: secondary progressive.

^a^ 5,141 (89.2%) of the individuals with unspecified phenotype were diagnosed before 1995.

^b^ 7,547 (43.7%) were diagnosed 1995‐2004.

To compare treatments with DMTs in DMSR and DNHMR, we considered treatments in DMSR that were ongoing or started from January 1, 2018, until the data extraction date (N = 18,168 treatments among 11,602 persons). We calculated the proportion of these treatments in the DMSR that could be identified in the DNHMR (N = 7230 treatments among 5944 persons). The comparison was conducted using personal ID, ATC codes, and treatment start date/prescription date (Table 4).

**TABLE 4:** Comparison of 18,168 treatments with DMTs in 11,602 persons in DMSR with DNHMR.

DMT	N (treatments) in 11,602 persons in DMSR	N (treatments) in 5,944 persons in DNHMR	Proportion of DMT treatments in DMSR recorded in DNHMR
Alemtuzumab	206	37	18.0%
Azathioprin	86	8	9.3%
Cladribine	455	113	24.8%
Dimethyl fumarate	3379	637	18.9%
Diroximel fumarate	66	9	13.6%
Fingolimod	1949	481	24.7%
Glatiramer acetate	1171	204	17.4%
Interferon beta‐1a	1104	179	16.2%
Interferon beta‐1b	60	9	15.0%
Methotrexate	107	<5	#
Mitoxantrone	8	0	0.0%
Mycophenolatmofetil	12	<5	#
Natalizumab	2156	1822	84.5%
Ocrelizumab	2330	2062	88.5%
Ofatumumab	796	317	39.8%
Ozanimod	54	10	18.5%
Peginterferon beta‐1a	381	48	12.6%
Rituximab	764	603	78.9%
Siponimod	38	<5	#
Teriflunomide	3046	682	22.4%
**TOTAL**	**18,168**	**7230**	**39.8%**

Abbreviations: DMT: Disease‐modifying treatment; DMSR: Danish Multiple Sclerosis Registry; DNHMR: Danish National Hospital Medication Register.

## 3 Results

### 3.1 Diagnoses of MS and Related Disorders in DMSR Versus DNPR

**FIGURE 1:**
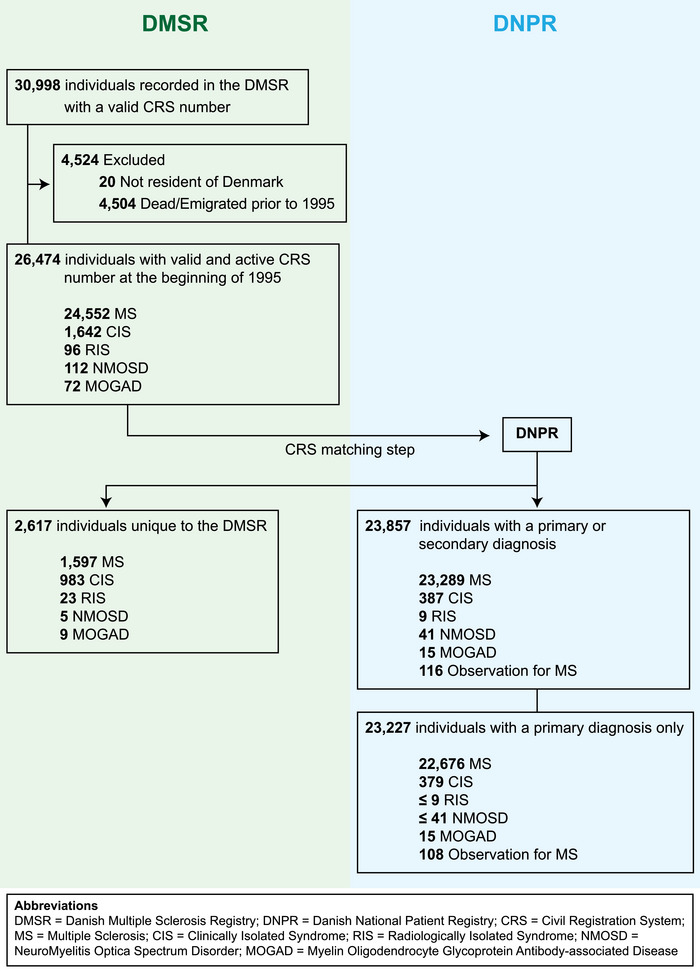
Flow chart towards the study population.

Figure 1 shows that of 30,998 persons recorded in DMSR with a valid CRS number, 4,524 were excluded due to death or emigration prior to January 1, 1995, or due to a CRS‐number indicating non‐residence in Denmark, leaving 26,474 persons in the study population with a diagnosis of MS (N = 24,552, 92.7%), CIS (N = 1642, 6.2%), NMOSD (N = 112, 0.4%), MOGAD (N = 72, 0.3%), or RIS (N = 96, 0.4%).

Table 2 shows that of the 26,474 persons, 2617 (9.9%) had no records of MS or related disorders in DNPR, while 23,857 persons (90.1%) could be identified in the DNPR with a primary or secondary diagnosis of MS or a related disorder. In total, 23,227 of the 26,474 persons (87.7%) were recorded with a primary diagnosis of MS or a related disorder in DNPR. Of the 2617 persons not recorded with MS or a related disorder in DNPR, most were diagnosed prior to 1995 (N = 2266, 86.6%).

Table 3 shows that of the 24,552 persons with MS in the DMSR, 1,597 (6.5%) had no records of MS in DNPR, while 22,676 (92.4%) had a primary MS diagnosis in DNPR. Among those not recorded in the DNPR (N = 1597), 92.5% (N = 1477) were diagnosed before 1995. The median age at the date of MS diagnosis was 39.3 years (IQR: 30.7; 48.2) based on DMSR data and 43.6 years (IQR: 34.0; 53.3) based on the first recorded MS primary diagnosis in DNPR.

In total, 37.6% (N = 9220 persons) had a clinical onset date before 1995. Diagnostic delay, expressed as median time from clinical onset to MS diagnosis was 2.0 (IQR: 0.4; 6.0) years. Median age at clinical onset was 34.3 (IQR: 26.8; 43.1) years, and median disease duration (time from clinical onset to the earliest of emigration, death, or data extraction) was 20.4 years (11.4; 31.0). Since the DNPR lacks data on clinical onset, these characteristics cannot be assessed using DNPR data alone.

A considerable proportion (23.5%) of persons with MS had no specified disease phenotype in DMSR. However, most (89.2%) of those with an unspecified phenotype in DMSR were diagnosed before 1995. After 2005, only 1.8% of persons with MS have no specification of the MS phenotype at the date of diagnosis. In contrast, 76.2% of MS cases in the DNPR lacked a specified phenotype.

### 3.2 Treatments With DMTs in DMSR Versus DNHMR

Table 4 provides an overview of the treatments with DMTs recorded in DMSR compared to those identified in the DNHMR. From January 1, 2018, to the date of data extraction, 18,168 treatments with DMTs in 11,602 persons were recorded in the DMSR. Of these, 7230 treatments (39.8%) involving 5944 individuals were identified in the DNHMR. The proportion of DMTs identified in DNHMR varied across different therapies, ranging from 0.0% (mitoxantrone) to 88.5% (ocrelizumab).

**FIGURE 2:**
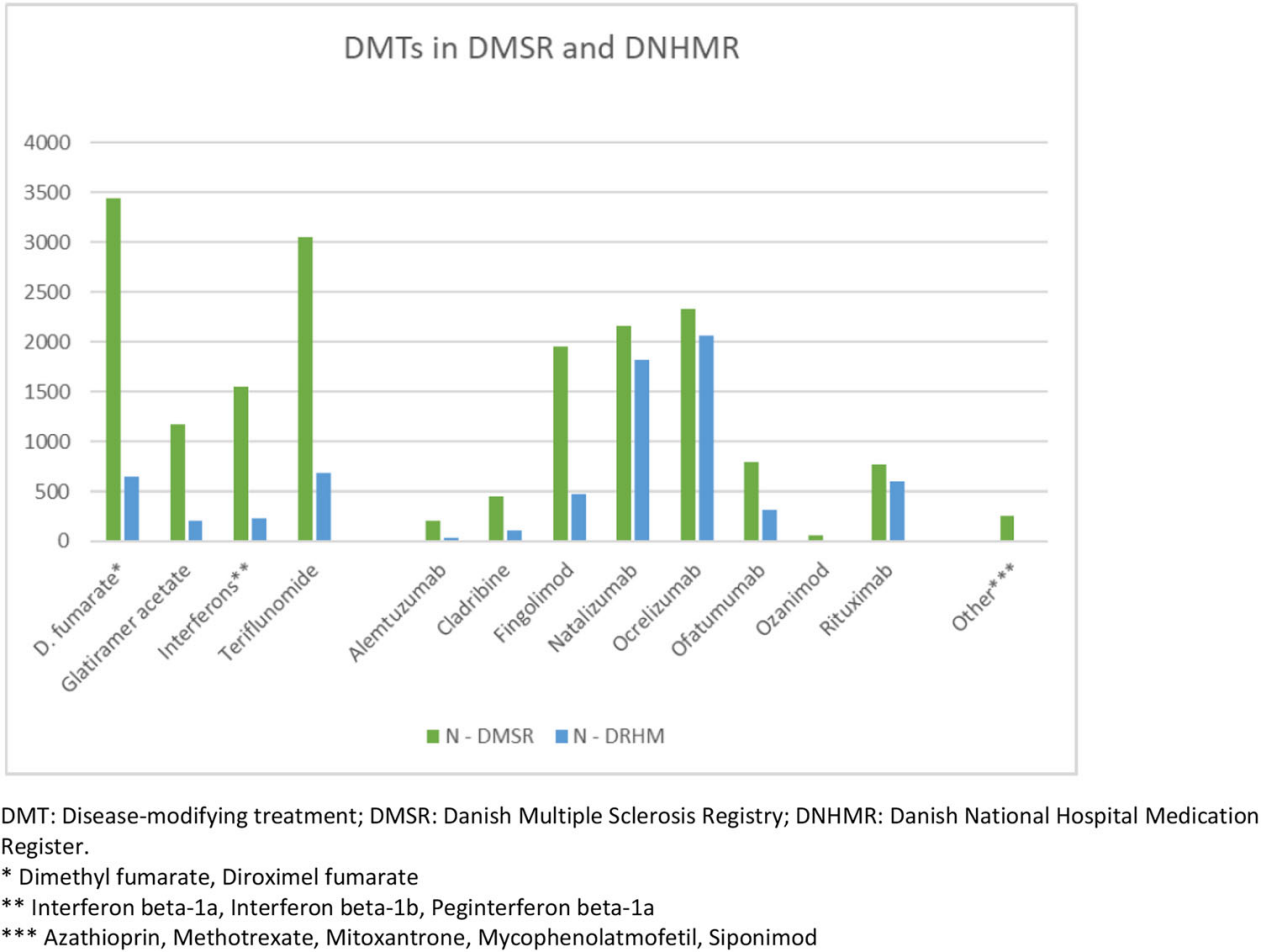
Number of treatments with DMTs in DMSR (18,168 treatments, 11,602 persons) versus DNHMR (7230 treatments, 5944 persons). DMT: Disease‐modifying treatment; DMSR: Danish Multiple Sclerosis Registry; DNHMR: Danish National Hospital Medication Register. * Dimethyl fumarate, Diroximel fumarate. ** Interferon beta‐1a, Interferon beta‐1b, Peginterferon beta‐1a. *** Azathioprin, Methotrexate, Mitoxantrone, Mycophenolatmofetil, Siponimod.

Among the 10,938 treatments with DMTs recorded in DMSR but not identified in the DNHMR, 2,609 (23.9%) were discontinued in 2018 and 4896 (44.8%) remained ongoing by November 2023, according to DMSR. Results are further visualized in Figure 2.

## 4 Discussion

This study described and compared three Danish nationwide registries core to MS research and highlighted key differences between them, underscoring the strengths and limitations of each registry for MS research.

A comparison of DMSR and DNPR revealed that 7.6% of persons with MS in DMSR lacked a primary MS diagnosis in DNPR, with most of these cases diagnosed before 1995. DNPR's limited capture of MS diagnoses before 1995 is likely due to the fact that outpatient contacts have only been included since 1995, and most MS‐related care involves outpatient visits. DMSR does not distinguish between inpatient and outpatient contacts. The lower median age at the time of diagnosis in DMSR compared to DNPR reflects earlier foundation and broader data capture of DMSR. Phenotype recording has improved significantly in the DMSR after 2005, with only 1.8% of cases diagnosed after 2005 lacking a specified phenotype. However, the proportion of persons with an unspecified phenotype is high both in DNPR (76.2%) and in DMSR (23.5%).

The comparison of DMSR and DNHMR regarding DMTs further demonstrated that DNHMR is not yet complete. Only 40% of DMT treatments documented in DMSR were identified in DNHMR. Further, DNHMR lacks exact dates of treatment discontinuation, and since DNHMR is a recent registry from 2018, it does not include treatments with DMTs from 1996 to 2018. This gap precludes its use to investigate the efficacy and safety of DMTs administered before 2018.

Each registry offers distinct advantages for research. The DMSR is most suitable for disease‐specific research, providing detailed data on MS onset and diagnosis, disease activity, and progression. It enables focused research studies on MS treatment efficacy, disease trajectories, and long‐term outcomes in persons with MS. While its primary focus is on MS and related conditions, it offers valuable insights into these areas and can be further enhanced by linking with other nationwide Danish registries to explore other characteristics, such as comorbidities and socioeconomic milestones.

DNPR can serve as a data source for broader epidemiological studies involving various health conditions from health registries. While it may not provide clinical characteristics needed for in‐depth MS research, it is highly effective for studies focusing on the identification of diagnoses. These findings highlight the complementary roles of these registries in MS research and emphasize the importance of integrating data from multiple data sources to enhance the scope and depth of epidemiological and clinical studies.

## 5 Conclusion

DMSR is suitable for disease‐specific research addressing treatment efficacy, disease development, and long‐term outcomes. In contrast, DNPR is well suited for broad epidemiological studies involving various health conditions and hospital utilization. The DNHMR will, once matured, be useful for studies involving medical treatment and comedication in hospitals. However, as each registry has limitations, for most studies the most suitable approach will be combining registries, depending on the research question.

## Author Contributions

**Hanna Joensen**: conceptualization, data curation, formal analysis, methodology, writing – original draft, writing – review and editing. **Elisabeth Framke**: conceptualization, methodology, writing – original draft, writing – review and editing. **Luigi Pontieri**: writing – review and editing. **Melinda Magyari**: conceptualization, methodology, writing – review and editing.

## Funding

The authors have nothing to report.

## Conflicts of Interest

HJ, EF, and LP have nothing to disclose. MM has served on the scientific advisory board for Sanofi, Novartis, Merck, and Moderna and has received honoraria for lecturing from Biogen, Merck, Novartis, Roche, Sanofi, Moderna, and Neuroxpharm.

## Ethics Statement

In Denmark, studies based on registry data only do not require approval from an ethics committee and neither require consent to participate nor consent for publication.

## Data Availability

The data underlying this article cannot be shared publicly due to data protection regulation. All data are stored in a protected server at the Danish Health Data Authority and can be accessed only by researchers authorized by the Danish Health Data Authority and approved by the Danish Multiple Sclerosis Registry board.
